# 

**Published:** 1888-06

**Authors:** 


					﻿io cts. a copy
JUIE, 1888.
$1.00 per 5 ear.
HALLS*
gj0VRN.AI / J
HEALTH
ESTABLISHED 18 5 4.
U°i\35
<#>-• 6
OFFICE: 206 BBOADWAY, EVENING POST BUTT.DING, Boom 11. N. Y.
Entered as Second-class Matter at the Post-Office, New York.
				

